# Clinical Characteristics and Outcomes of COVID-19 Acute Respiratory Distress Syndrome Patients Requiring Invasive Mechanical Ventilation in a Lower Middle-Income Country

**DOI:** 10.2478/jccm-2021-0044

**Published:** 2022-02-09

**Authors:** Taymmia Ejaz, Fazal Rehman, Arslan Ahmed, Safia Akhlaq, Sheema Saadia, Adil Aziz, Erfan Hussain

**Affiliations:** 1 The Aga Khan University Hospital, Karachi, Pakistan

**Keywords:** ARDS, COVID-19, mechanical ventilation, mortality

## Abstract

**Background:**

COVID-19 related acute respiratory distress syndrome (ARDS) requires intensive care, which is highly expensive in lower-income countries. Outcomes of COVID-19 patients requiring invasive mechanical ventilation in Pakistan have not been widely reported. Identifying factors forecasting outcomes will help decide optimal care levels and prioritise resources.

**Methods:**

A single-centre, retrospective study on COVID-19 patients requiring invasive mechanical ventilation was conducted from 1st March to 31st May 2020. Demographic variables, physical signs, laboratory values, ventilator parameters, complications, length of stay, and mortality were recorded. Data were analysed in SPSS ver.23.

**Results:**

Among 71 study patients, 87.3% (62) were males, and 12.7% (9) were females with a mean (SD) age of 55.5(13.4) years. Diabetes mellitus and hypertension were the most common comorbidities in 54.9% (39) patients. Median(IQR) SOFA score on ICU admission and at 48 hours was 7(5-9) and 6(4-10), and median (IQR) APACHE-II score was 15 (11-24) and 13(9-23), respectively. Overall, in-hospital mortality was 57.7%; 25% (1/4), 55.6% (20/36) and 64.5% (20/31) in mild, moderate, and severe ARDS, respectively. On univariate analysis; PEEP at admission, APACHE II and SOFA score at admission and 48 hours; Acute kidney injury; D-Dimer>1.5 mg/L and higher LDH levels at 48 hours were significantly associated with mortality. Only APACHE II scores at admission and D-Dimer levels> 1.5 mg/L were independent predictors of mortality on multivariable regression (p-value 0.012 & 0.037 respectively). Admission APACHE II scores, Area under the ROC curve for mortality was 0.80 (95%CI 0.69-0.90); sensitivity was 77.5% and specificity 70% (cut-off ≥13.5).

**Conclusion:**

There was a high mortality rate in severe ARDS. The APACHE II score can be utilised in mortality prediction in COVID-19 ARDS patients. However, larger-scale studies in Pakistan are required to assess predictors of mortality.

## Introduction

Coronavirus disease 2019 (COVID-19) pandemic, caused by Severe Acute Coronavirus 2 (SARS CoV-2) has spread globally. Over a hundred million people have been infected, with over 2.5 million deaths by the end of February 2021[1]. During the early period of the pandemic, studies from China indicated that most patients stay asymptomatic; however, 20% of patients follow a more severe course, and one-fourth of them require admission to an intensive care unit (ICU)[2]. Subsequent data have substantiated that disease severity, need for mechanical ventilation, and mortality varies considerably between different countries in different studies[3].

The most severe complication of COVID-19 is acute respiratory distress syndrome (ARDS), resulting in hypoxic respiratory failure[4]. The severity of hypoxia classifies the ARDS into mild, moderate and severe categories [5]. ARDS generally requires mechanical ventilation, but in the case of COVID-19, the outcome is not well known[6]. In addition, much variability has been observed in the clinical presentation and clinical course of COVID-19 associated ARDS, highlighting the importance of developing different management plans[7,8].

Various factors have been identified linked to the severity of disease and the likelihood of developing ARDS and related adverse outcomes. These factors include male sex, age more than 60 years, obesity, comorbidities such as diabetes mellitus, hypertension, previous lung disease, and specific laboratory parameters such as low lymphocyte count and high D-dimer levels[9].

There are local studies from Pakistan which have evaluated the clinical presentation, laboratory parameters and factors determining the outcome in patients with Severe COVID-19. However, there are no studies on invasive mechanical ventilation in COVID-19 ARDS patients from Pakistan [10, 11, 12].

The present study was undertaken to evaluate presentations, laboratory parameters, outcomes and the factors determining the outcome among COVID-19 ARDS patients requiring invasive mechanical ventilation; thereby, disseminating a better understanding of this dreadful complication of COVID-19 in one Pakistani population.

## Materials and Methods

This was a retrospective, observational study undertaken in the Department of Medicine, The Aga Khan University Hospital, Karachi, Pakistan.

Ethical approval was taken from the Institutional Review Board (ERC Number: 2020-4796-10703.). Data collection was done by retrospectively reviewing files and electronic health records of all patients with SARS-CoV-2 RT-PCR who presented with a positive result from nasopharyngeal or tracheal aspirate and were placed on invasive mechanical ventilation between 1^st^ March to 31st May 2020.

Data collection was done on demographic variables including age, gender, body mass index (BMI), co-morbid conditions. The level of care at admission was also recorded and resulted in patients being either direct admission to the ICU’s special acute care unit (SCU), which is equivalent to High-Dependency Units (HDU) or to a ward.

Laboratory test results obtained on ICU admission and after 48 hours were recorded.

Test parameters included haemoglobin (g/dl), white cell count (x10^9/L), platelet count (x10^9/L), blood urea nitrogen(mg/dl), creatinine(mg/dl), sodium levels (mmol/L), potassium (mmol/L), bicarbonate(mmol/L), C-reactive protein (mg/L), serum ferritin (ng/ml), D-Dimer levels (mg/ml), procalcitonin (ng/ml), Lactate dehydrogenase (LDH I.U/L), serum lactate(mmol/L), troponin-I (ng/ml), arterial pH, arterial partial pressure of carbon dioxide (PaC02 mmHg) and arterial partial pressure of oxygen (Pa02 mmHg).

The following physical signs were recorded on ICU admission and at 48 hours; pulse rate, mean arterial pressure, GCS (Glasgow Coma Scale) and respiratory rate and temperature, as were ventilator parameters such as ventilation mode, positive end-expiratory pressure (PEEP), the fraction of inspired oxygen (FiO_2_), PaO2/FiO2 ratio, compliance and tidal volume (ml/ kg).

Use of proning, sedation, neuromuscular blockade, vasopressors, and inotropes were reviewed. Sequential organ failure assessment score (SOFA) and Acute Physiologic Assessment and Chronic Health Evaluation II (APACHE II) scores were calculated at ICU admission and at 48 hours of stay[13,14]. Additionally, outcome measures including in-hospital mortality, ICU mortality, ICU length of stay, length of hospital stay and days on invasive mechanical ventilation were noted.

Time from symptom onset to ER presentation and symptom onset to ICU admission were determined, and code status changes during admission up to the withdrawal of care or Do-Not-Resuscitate (DNR) code were also recorded.

Complications include septic shock, myocardial injury, acute kidney injury, need for renal replacement therapy, hospital-acquired/ventilator-associated pneumonia, critical illness myopathy/ neuropathy, bedsores, ICU delirium, gastrointestinal bleed, and barotrauma others were noted.

### Definitions

According to the Berlin definition, ARDS was categorised as mild, moderate and severe categories[5]. According to the Kidney Disease: Improving Global Outcomes guidelines (KDIGO) [15]. Myocardial injury was diagnosed based on elevated biomarkers above the 99^th^ percentile or new electrocardiographic and/or echocardiographic findings. Septic shock was defined according to the Third International Consensus Definitions for Sepsis and Septic Shock (Sepsis-3) guidelines [16]. Hospital-acquired and Ventilator-associated pneumonia was defined according to the American Thoracic Society guidelines [17].

### Data analysis

Data analysis was prepared using the Statistical Package for the Social Sciences (SPSS) version 23. Continuous variables were presented as the mean (SD) or median with interquartile ranges (IQR) where appropriate. Categorical data were presented as the frequency (n) and the percentages (%). For comparison of variables between survivors and non-survivors, the Chi-squared or the Fischer’s exact test were used for categorical variables, and the Mann-Whitney U test or Student t-test were used for continuous variables where appropriate.

The significance level was set at an alpha value, 0.05.

Univariate analysis was done, and the odds ratio for mortality was determined by binary logistical regression. For multi-variate analysis, variables with a statistically significant p-value (<0.05) were used; only six variables were selected for the regression model due to the small sample size.

The Area under the curve (AUC) using receiver operating characteristic (ROC) was calculated for APACHE II and SOFA scores models.

## Results

A total of 71 patients were included in the study. Mean (SD) age was 55.5 (13.4) years, 87.3% (62) were males and 12.7% (9) were females. Mean (SD) body mass index (BMI) was 26.4 (3.6) kg/m2.

Associated co-morbid conditions were present in 73% (51) of patients; diabetes and hypertension were the most common among them and were present in 54.9% (39) (Table 1).

**Table 1:** Demographic and clinical features of the study population

Variables Median (Interquartile ranges)	Survivors (n=30)	Non-Survivors (n=41)	All patients (n=71)	P-value
Age (years)	55(47.5-64)	59(46-65)	56(48-64)	0.35
Age (years)				0.86
<40	41.7%(5)	58.3%(7)	16.9%(12)	
41-50	55.6%(5)	44.4%(4)	12.7%(9)	
51-59	41.2%(7)	58.8%(10)	23.9%(17)	
>60	39.4%(13)	60.6%(20)	46.5%(33)	
Diabetes Mellitus	38.5%(15)	61.5%(24)	54.9%(39)	0.47
Hypertension	41%(16)	59%(23)	54.9%(39)	0.81
Ischemic heart disease	41.7%(10)	58.3%(14)	33.8%(24)	0.94
Chronic kidney disease	33.3%(3)	66.7%(6)	12.7%(9)	0.72
Proning	43.9%(18)	56.1%(23)	57.7%(41)	0.74
Vasopressors requirement	37.5%(15)	62.5%(25)	56.3%(40)	0.35
BMI (kg/m2)	29.5 (24.04-28.53)	25.95 (24.04-28.5)	25.95 (24.22-27.68)	0.7
ICU Admission SOFA score	5(4-7)	12(9-14)	7(5-9)	<0.001
48 hours SOFA score	4(2-6)	10(9-12)	6(4-10)	<0.001
ICU Admission APACHE II score	8(7-10)	21(14-28)	15(11-24)	<0.001
APACHE II 48 score hours	9 (6- 11)	20 (12-25)	13(9-23)	<0.001
PEEP ICU Admission	8(8-10)	8(6-10)	10(8-10)	0.217
PEEP 48 hours	10(8-11)	10(8-12)	10(7.75-12)	0.007
Tidal volume (ml/kg) at ICU admission	6(5.62-6.43)	6.42(5.62-6.84)	6.17(5.62-6.57)	0.674
Tidal volume at 48 hours	6.35(5.9-6.86)	6.13(5.62-6.85)	6.27(5.7-6.85)	0.548
PF ratio ICU admission (PA02/Fi02)	118(81.09-219.31)	107.37(65.75-141.25)	112.5(76.75-160)	0.041
PF ratio 48 hours(PA02/Fi02)	164.7(115.03-221.41)	141.41(108.03-192.75)	154.55(108.75-199.25)	0.208
Duration of symptom onset to ER	7(4-10)	7(5-10)	7(4-10)	0.313
presentation				
Symptom onset to ICU admission	9(5.7-11.25)	9(7-12)	9(7-12)	0.472
Length of ICU stay	6(4-9)	8(4-11.5)	8(4-10)	0.177
Length of ICU stay with IMV	4.5(3-7)	8(4-11)	6(3-10)	0.008
The total length of stay	17(10.75-21.25)	9(7.5-15)	11(8-20)	0.001
Acute kidney injury	66.6%(20)	90.2%(37)	80.3%(57)	0.014

* Values as Median (Interquartile ranges); p-value calculated by Chi-square or Fischer exact for categorical variables and Mann–Whitney U, or independent sample t-test for continuous variables where applicable. BMI: Body mass index, SOFA: Sequential organ failure assessment score, APACHE II: Acute Physiologic Assessment and Chronic Health Evaluation II, PEEP: positive end-expiratory pressure, IMV: Invasive mechanical ventilation.

Other uncommon conditions were obstructive airway disease in 4.2% (3) of patients and a history of cerebrovascular accident in 2.8% (2). Median positive end-expiratory pressure (PEEP) on admission to the ICU admission and at 48 hours was 10 (IQR 8-10) and 10 (IQR 7.75-12) cm H20, respectively. Proning was carried out on 57.7% (41) of patients, 56.3% (40) required vasopressor support and 11.3% (8) required inotrope support. Among patients requiring vasopressor support, 65%% (26/40) received norepinephrine alone, whereas 35% (14/40) received a norepinephrine-vasopressin combination. The mean (SD) SOFA scores at ICU admission and 48 hours were 7.32 (2.9) and 7.14 (4.3), respectively. Mean (SD) APACHE-II scores at ICU admission and 48 hours was 17.4(8.1) and 15.8 (8.9), respectively (Table 1).

The in-hospital mortality rate was 57.7% (41); ICU mortality rate was 50.7%v (36); among the survivors, 26 were discharged, and four left against medical advice. A decision to withdraw mechanical ventilation was made in 42.3% of cases (30). Of these, 26 (86.7%) died, 21(80.8%) in the ICU, and five of this cohort (19.2%) were transferred out of the ICU.

The mortality rate was 25% (1/4), 55.6% (20/36) and 64.5% (20/31) among patients with mild, moderate, and severe ARDS, respectively. The majority of patients, 52.1% (37), were directly admitted to an ICU, whereas 45.1% (32) were initially admitted to a special care unit and later transferred to an ICU. The mortality rate was 62.2% (23/37) among those admitted directly to ICU from ER and 50% (16/32) among patients who were transferred from SCU to ICU.

There was no statistically significant association between age, gender, proning, and vasopressor requirement and mortality. Length of hospital stay was significantly higher among survivors. PEEP requirement at 48 hours was significantly higher among non-survivors. Of non-survivors, 90.2% (31/41) had acute kidney injury and was associated with mortality on univariate analysis.

### Laboratory parameters

Non-survivors had higher D-Dimer levels at 24 hours and 48 hours after ICU admission (p-value 0.028 and 0.045); ferritin (p-value =0.15), lactate dehydrogenase (p-value=0.40) and pro-calcitonin levels(p-value=0.41) at the time of admission or within 24 hours of admission were not significantly different. Ferritin levels at 48 hours were significantly different (p-value 0.008), higher lactate dehydrogenase levels were recorded at 48 hours (p-value 0.004), and non-survivors also had higher pro-calcitonin levels at 48 hours. However, C- reactive protein levels (p-value= 0.054) were not significantly different. Differences in laboratory parameters among survivors and non-survivors are given in Table 2.

**Table. 2:** Laboratory parameters among survivors and non-survivors

	Survivors (n=30)	Non-Survivors (n=41)	All patients (n=71)	p-value
Hemoglobin at 24 hours ( g/dl) N=71	13.05(11.5-13.5)	11.9(9.85-14)	12.4(10.6-13.7)	0.34
Hemoglobin at 48 hours( g/dl) N=70	12.05(10.7-12.9)	11.05(9.45-13.15)	11.75(9.97-12.97)	0.112
TLC at 24 hours (x10^9/L)	11.15(8.0-15.1)	14.3(9.3-18.2)	12.9(8.9-16.5)	0.046
TLC at 48 hours (x10^9/L)	11.15(8.5-14.9)	13.95(9.3-20.0)	12.6(12.6-16.07)	0.133
Platelets count at 24 hours(x10^9/L)	3310(237.25-363)	224(157.5-335.5)	269(198-353)	0.063
Platelets count at 48 hours(x10^9/L)	334.5(277.5-406.7))	212.5(132.75-317.5)	269.5(171.25-356.25)	<0.001
Blood Urea Nitrogen(mg/dl)	25(15.75-43)	27(22.5-51.5)	26(19-43)	0.159
Blood Urea Nitrogen at 48 hours( mg/dl)	26(21-49.25)	37.5(28.25-58.25)	35.5(22.75-54.25)	0.057
Creatinine at 24 hours ( mg/dl)	1.25(0.9-1.65)	1.4(1.1-2.05)	1.4(1-1.9)	0.111
Creatinine at 48 hours(mg/dl)	1(0.7-1.35)	1.85(0.9-25)	1.3(0.8-2.35)	0.007
Sodium 24 hours (mmol/L)	138.5(131.75-141)	139(133-143)	139(133-142)	0.307
Sodium 48 hours(mmol/l)	141(136.75-144)	143(139.25-147.75)	142.5(138-147)	0.032
Bicarbonate 24 hours (mmol/L)	22.3(19.3-24.8)	20.7(17.5-23.45)	21.9(18-24)	0.102
Bicarbonate at 48 hours(mmol/L)	22.8(21.2-26.8)	20.65(18.75-22.5)	21.55(19.9-24.5)	0.002
C-Reactive Protein 24 hours (n=70) (mg/L)	160(69-236)	165(58-203.5)	161.5(63-214.5)	0.948
C-Reactive Protein at 48 hours (n=63) (mg/L)	123(48.5-196)	192.5(85.5-278)	165(61-244)	0.054
C-Reactive Protein Max (n=60) (mg/L)	201(169-292)	235(162-317.5)	230(169.25-301)	0.6
Ferritin at 24 hours (n=68) (ng/ml)	879.5(420.75- 2518.5)	1519(669.75-3183.5)	1278.5(548.75-2566)	0.159
Ferritin at 48 hours (n=53) (ng/ml)	674(304-1883.5)	2075(896.5-3000.5)	1287(599.5-2752.75)	0.008
Ferritin Max (n=61) (ng/ml)	1169.5(648-4424)	2282(1193.5-6727)	1923(758.5-5415)	0.088
D-Dimer at 24 hours (n=69) (mg/ml)	1.8(1.05-4)	3.65(2.1-13.72)	3.3(1.25-9.5)	0.038
D-Dimer at 48 hours (n=60) (mg/L )	3.2(1.7-11.6)	8.1(2.35-15.4)	5.3(1.95-15.3)	0.045
D-Dimer Max(n=61) (mg/L )	5.3(2.5-16.8)	18.3(6.5-30)	14.5(4.6-30)	0.009
Procalcitonin (n=68) (ng/ml)	0.53(0.209-1.45)	0.80(0.17-3.58)	0.603(0.202-2.102)	0.411
Procalcitonin at 48 hours (n=54) (ng/ml)	0.51(0.25-2.99)	2.85(0.48-9.72)	1.33(0.431-7.47)	0.077
LDH 24 hours (n=67) (I.U/L)	650(535-774.5)	667.5(569.75-867)	652(569-831)	0.40
LDH48(n=58) (I.U/L)	575(430-696.75)	670(579.25-823)	618(498-743.5)	0.004
pH at admission	7.4(7.35-7.43)	7.37 (7.25-7.43)	7.39(7.32-7.43)	0.21
pH 48 hours	7.41(7.36-7.45)	7.34 (7.27-7.41)	7.39(7.315-7.43)	0.001

*Values as Median (Interquartile ranges); p-value calculated by Chi-square or Fischer exact for categorical variables and Mann–Whitney U, or independent sample t-test for continuous variables where applicable. ¶ Maximum laboratory value during the hospital stay. TLC: Total leukocyte count; LDH: Lactate Dehydrogenase.

### Complications:

Acute kidney injury was the most common complication reported in 80.3% (57) of patients (Table 3). The mortality rate was 64.9% (37/57) in patients with acute kidney injury and 28.6% ( 4/14) without acute kidney injury. Renal replacement therapy was required in 22.5% (16/71) of patients, and 75% (12/16) of those requiring renal replacement therapy died. Superimposed infections were frequent; culture-proven bacterial infections were recorded in 53.5% (38) patients. Cardiopulmonary resuscitation with the return of spontaneous circulation was achieved in 16.9% (12/71) of the patients; 58.3% (7) among these had their code status to Do-Not-Resuscitate order or withdrawal of support. None of these patients survived, and survival to discharge was 0 of 12 among these patients.

**Table 3:** Complications observed in patients during the hospital stay

Complications	Frequency	Percentage(%)
Septic Shock	44	62.0
Multi-organ Dysfunction	31	43.7
Myocardial injury	30	42.3
Thrombotic complications	2	2.8
Acute kidney Injury	57	80.3
Renal replacement therapy	16	22.5
CRRT		2.8
Both CRRT/ Hemodialysis		8.5
Intermittent Hemodialysis		11.3
Hospital-acquired Pneumonia/VAP.	38	53.5
Acinetobacter	14	
Stenotrophomonas		
Pseudomonas		
Klebsiella		
E.coli		
Fungal infections	28	39.4
Bacteremia	13	18.3
Catheter-associated urinary tract infection	8	11.3
Pneumothorax	11	15.5
Subcutaneous emphysema	9	12.7
Critical illness myopathy	5	7.2
Bed scores	2	2.9
GI Bleed	17	24.6
Re-intubation	4	5.8
Arrhythmias(new-onset)	20	28.1
CPR with ROSC	12	16.9
Diabetic ketoacidosis	6	8.45

Abbreviations: CRRT: Continuous renal replacement therapy, VAP: Ventilator-associated pneumonia, CPR: Cardiopulmonary resuscitation, ROSC: Return of spontaneous circulation

### Predictors of mortality

PEEP, APACHE II and SOFA scores on admission and at 48 hours are given in Table 4. The presence of acute kidney injury (p-value=0.019), D-Dimer>1.5 mg/L (p-value=0.031) and higher lactate dehydrogenase levels at 48 hours (p-value=0.017) were significantly associated with mortality.

**Table 4:** Univariate analysis of predictors of mortality

Variable	p-value		Odds ratio (95% Confidence interval)
Age	0.52		1.01 (0.97-1.04)
Age>60	0.65		1.24 (0.48-3.21)
PEEP at 48 hours	0.009		1.3 (1.06-1.6)
PEEP at 24 hours	0.32		1.09 (0.91-1.32)
PF ratio admission	0.032		0.99 (0.98-0.99)
PF ratio at 48 hours	0.56		0.99 (0.99-1.003)
APACHE-II at 24 hours	0.000		1.19 (1.08-1.31)
APACHE-II 48 hours	0.000		1.17 (1.07-1.27)
SOFA score at 24 hours	0.000		1.48 (1.19-1.85)
SOFA score at 48 hours	0.000		1.47 (1.2-1.8)
Hypertension	0.81		1.11 (0.434-2.87)
Acute kidney injury	0.019		4.62 (1.28-16.64)
Myocardial injury	0.416		1.49(0.56-3.90)
TLC at 24 hours (x10^9/L	0.079		1.06 (0.99-1.14)
Platelets count at 48 hours(x10^9/L)	0.001		0.99 (0.98-099)
Dimer>1.5 mg/L	0.031		3.1 (1.11-8.71)
D-Dimer Maximum	0.017		1.06 (1.01-1.12)
D-Dimer at 24 hours (n=69) (mg/ml)	0.067		1.05 (0.996-1.12)
D-Dimer at 48 hours (n=60) (mg/L )	0.089		1.05 (0.99-1.12)
C-Reactive Protein 24 hours (n=70)(mg/L)	0.923		1 (0.996-1.005)
C-Reactive Protein >180 mg/L	0.80		1.12 (0.43-2.9)
Procalcitonin >1 ng/ml	0.236		1.82 (0.67-4.93)
LDH 24 hours (n=67) (I.U/L)	0.212		1.001 (1.00-1.002)
LDH 48 hours (I.U/L)	0.017		1.005(1.001-1.008)

Abbreviations: SOFA: Sequential organ failure assessment score, APACHE II: Acute Physiologic Assessment and Chronic Health Evaluation II, PEEP: positive end-expiratory pressure, TLC: Total leukocyte count; LDH: Lactate Dehydrogenase. *p-value calculated by Chi-square or Fischer exact for categorical variables and *Mann*–*Whitney U*, or independent sample t-test for continuous variables where applicable.

Only APACHE II score 0n admission, and D-Dimer levels> 1.5 mg/L were found to be independent predictors of mortality on multivariable regression analysis (Table 5).

**Table 5:** Multi-variate regression analysis of predictors of mortality

Variable	p-value	Odds ratio (95% Confidence interval)
SOFA score Admission	0.26	1.19(0.8-1.64)
APACHE-II score Admission	0.012	1.18(1.03-1.34)
PF (PA02/Fi02) ratio Admission	0.20	0.99(0.98-1.003)
Dimer>1.5 mg/L	0.037	5.15(1.108-23.96)
Acute kidney injury	0.75	0.76(0.13-4.26)
PEEP at admission (cmH20)	0.26	1.17(0.88-1.57)

Abbreviations: SOFA: Sequential organ failure assessment score, APACHE II: Acute Physiologic Assessment and Chronic Health Evaluation II, PEEP: positive end-expiratory pressure

**Fig. 1:**
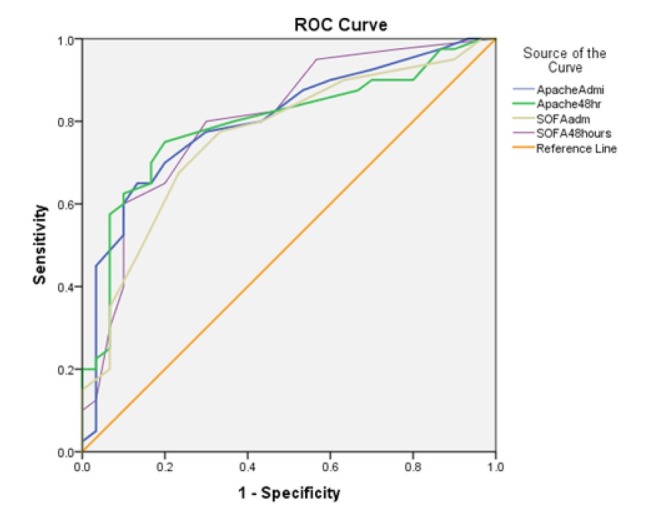
Comparison of the Area under the ROC curve between SOFA and APACHE II scoring models

Admission APACHE II scores on ICU admission (p-value <0.001) and SOFA scores at 48 hours (p-value <0.001) showed similar areas under the ROC curve and were statistically significant. (Figure 1)

However, SOFA scores at 48 hours showed the largest Area Under the Curve and were more sensitive in predicting mortality (Table 6).

**Table 6:** Area under ROC curve and cut-off values of APACHE II and SOFA score for prediction of mortality

Scoring variable	Area under ROC curve (95% confidence interval)	Standard error	p-value	Cut-of value	Sensitivity	Specificity
Admission APACHE II score	0.803(0.697-0.908)	.054	0.000016	13.5	0.775	0.70
48 hour APACHE II score	0.795(0.687-0.903)	.055	0.000027	12.5	0.75	0.80
Admission SOFA score	0.763(0.649-0.876)	.058	0.000181	6.5	0.775	0.67
48 hour SOFA score	0.805(0.699-0.910)	.054	0.000014	5.5	0.80	0.70

Abbreviations:ROC: Receiver operating curve, SOFA: Sequential organ failure assessment score, APACHE II: Acute Physiologic Assessment and Chronic Health Evaluation II

## Discussions

In this initial pandemic era, data of mechanically ventilated COVID-19 ARDS patients in Pakistan mortality rate was high.

The presently reported study population also had a high proportion of patients with severe ARDS, and mortality varied with ARDS severity. In a meta-analysis of 69 studies on case fatality rates in COVID-19 patients requiring invasive mechanical ventilation, the actual case fatality rate in patients with known outcomes was 56% and ranged from 43% to 64% [18]. Mortality rates in the epicentres during the earlier pandemic period were also high. ICU mortality rate was 25.7% in a systematic review of 15 studies on critically ill ICU patients from March till May 2020; however, 56.1% of the patient population was still hospitalised in an ICU at the time of publication [19]. A high mortality rate of 97% was reported in Wuhan by Zhou et al. (2020 ) during the pandemic period from December to January 2020, [20], and Yang et al. (2020) reported an 81% mortality rate in patients requiring intermittent mandatory ventilation. [21] Wang et al. (2020) reported a mortality rate of 97% among mechanically ventilated patients [22]. Grasselli et al. (2020) reported an overall mortality rate of 26% among 1581 patients. However, 920 patients were still being treated in an ICU, and the mortality rate among patients with definite reported outcomes was 61.27%. [23].

In comparison, Gupta et al. (2020) reported a mortality rate of 39.5% in a multicenter centre study across 65 ICUs in the USA, although 6.2% were still hospitalised at the end of the study follow-up[25].

Another study reported the mortality among invasively ventilated patients in the UK as 56.8% [26].

King et al. (2020) reported a mortality rate of 42.7% among the mechanically ventilated population [27], and Auld et al. (2020) [28] reported a lower rate of 37.5%. Søvik et al. (2021 ) reported a significantly lower mortality rate of 13% among mechanically ventilated patients; however, only 25% of patients in the cohort had severe ARDS, in contrast to 64.5% in our study [29].

The mortality rate in our study and various other studies on invasively ventilated COVID-19 patients was also higher than that reported (35.3%) in the LUNG SAFE (2016) multicenter international cohort study. However, the study cohort had a higher population of patients with mild ARDS (30.0%) and moderate ARDS (46.6%) [30].

Hospital mortality was 34.9% in mild, 40.3% in moderate, and 46.1% in severe ARDS, whereas mortality rate was 25% (1/4), 55.6% (20/36) and 64.5% (20/31) among patients with mild, moderate and severe ARDS respectively in our study. Ferrando et al. (2020) reported lower mortality rates, 24% in mild, 29% in moderate and 39% in severe COVID-19 ARDS and found no differences in physiological parameters in COVID-19 and non-Covid 19 ARDS [31]. Our study population had a lower median PF (PA02/Fi02) ratio on admission, 112.5(76.75-160), and 43.7% (31) and 38% (27) of patients had a PF ratio of less than 200 and 100, respectively. Ferrando et al. (2020) reported an average PF ratio of 120 cmH20 [31].

The variation in ICU resources can explain the variation in mortality rate among various studies, the severity of ARDS, the severity of disease, age group, sicker patient population, the median time of symptoms to ICU admission, and pandemic phases study follow-up period. Most studies from the earlier pandemic period had patients still hospitalised in ICU, whereas disposition data for all patients were available in our study.

There was no statistically significant difference in the median age of survivors and non-survivors, although the mortality rate was 70% in patients >70 years of age in our study. This was in contrast to various other studies where age was an independent predictor of mortality [23].

ICU length of stay of survivors was similar to that reported by Ferrando et al. (2020) [31]; the median time from onset of symptoms to ICU admission was also comparable with other studies [21,25,31].

SOFA and APACHE II score has been evaluated for prognosis in COVID-19. Non-survivors had higher SOFA scores in a study by Auld et al. (2020) [28]; similarly, Wang et al. (2020) [32] and Cheng et al. (2021) [33] reported higher APACHE II scores. They found APACHE II scores a useful predictor of mortality; the reported AUC (0.90) was higher than our study; however, sensitivity was lower in comparison (61.9%). Zou et al. (2020 ) also found the APACHE II scores as an independent predictor of mortality in hospitalised patients, consistent with our study[34]. On the contrary, Isted et al. (2020) from the UK reported no differences among APACHE II scores among survivors and non-survivors [35]. Admission SOFA scores and admission D-Dimer was found to be independent predictors of mortality by Zhou et al. (2020) [20].

Hirsh et al. (2020) [36] and Fominseky et al. (2021 ) [37] reported acute kidney injury in 89.7% and 75% of patients requiring intermittent mandatory ventilation, respectively, a similar rate (80.3%) was observed in our study. Thakkar et al. (2020) reported acute kidney injury in 66.5% of ICU patients, renal replacement therapy was required in 50%, and mortality in patients requiring renal replacement therapy was 70%[38].

Among the 12 patients who achieved delayed return of spontaneous circulation in our study, survival to discharge was 0. Thapa et al. (2021 ) [39] also reported similar findings with a survival to discharge rate of 0. Sheth et al. (2020) reported a 100% mortality rate in patients with delayed return of spontaneous circulation [40]. Shao et al. (2020) reported a 30-days survival rate of 2.9% in Wuhan, where only one patient had a favourable outcome [41]. Hayek et al. (2020) reported slightly better outcomes, as, among patients receiving cardiopulmonary resuscitation, delayed return of spontaneous circulation was achieved in 135/400, and among the 135 patients, only 12%(48/135) survived to the point of hospital discharge [42].

There are several limitations of our study. First, this was a single centre, retrospective study with a sicker patient population as the median time to symptoms onset to an ICU admission was nine days (7-12). Some patients had missing laboratory parameters, and data on compliance and driving pressure was not collected. Finally, in a resource-constrained country such as Pakistan, the hospital in which this study was conducted is considered a well-structured tertiary care setup; hence results of the study cannot be generalised to other Pakistani patients.

## Conclusion

There was a high mortality rate in patients with severe ARDS. APACHE II score and D-dimer were reliable for predicting mortality; however, larger-scale studies are required to assess the predictors of mortality.
